# Clinical characteristics and outcome of patients with combined hepatocellular-cholangiocarcinoma—a European multicenter cohort

**DOI:** 10.1016/j.esmoop.2023.100783

**Published:** 2023-02-06

**Authors:** K. Pomej, L. Balcar, K. Shmanko, S. Welland, V. Himmelsbach, B. Scheiner, A. Mahyera, B. Mozayani, M. Trauner, F. Finkelmeier, A. Weinmann, A. Vogel, M. Pinter

**Affiliations:** 1 Division of Gastroenterology and Hepatology, Department of Internal Medicine III, Medical University of Vienna, Vienna; 2 Liver Cancer (HCC) Study Group Vienna, Division of Gastroenterology and Hepatology, Department of Internal Medicine III, Medical University of Vienna, Vienna, Austria; 3 Department of Internal Medicine I, University Medical Center of the Johannes Gutenberg University Mainz, Mainz; 4 Department of Gastroenterology, Hepatology and Endocrinology, Hannover Medical School, Hannover; 5 Department of Gastroenterology, Hepatology and Endocrinology, University Hospital Frankfurt, Frankfurt/Main, Germany; 6 Division of Cancer, Department of Surgery and Cancer, Imperial College London, Hammersmith Hospital, London, UK; 7 Department of Pathology, Medical University of Vienna, Vienna, Austria

**Keywords:** mixed hepatocellular-cholangiocarcinoma, hepato-cholangiocarcinoma, sorafenib, chemotherapy, immunotherapy, immune checkpoint inhibitor

## Abstract

**Background:**

There is no clear consensus on the optimal systemic treatment regimen in combined hepatocellular-cholangiocarcinoma (cHCC-CCA) patients. We describe clinical characteristics and outcome of cHCC-CCA patients, with a special focus on patients receiving palliative systemic therapy, including immune checkpoint inhibitors (ICIs).

**Methods:**

In this European retrospective, multicenter study, patients with histologically proven cHCC-CCA treated at four institutions between April 2003 and June 2022 were included. In patients receiving palliative systemic therapy, outcome was compared between cytotoxic chemotherapy (CHT)- and non-cytotoxic CHT (nCHT)-treated patients.

**Results:**

Of 101 patients, the majority were male (*n* = 70, 69%) with a mean age of 64.6 ± 10.6 years. Only type of first-line treatment was independently associated with overall survival (OS). Palliative systemic therapy was administered to 44 (44%) patients. Of those, 25 (57%) patients received CHT and 19 (43%) had nCHT (*n* = 16 of them sorafenib) in systemic first line. Although there was no significant difference in overall response rate (ORR; CHT versus nCHT: 8% versus 5%), disease control rate (24% versus 21%), and median progression-free survival {3.0 months [95% confidence interval (CI) 1.4-4.6 months] versus 3.2 months (95% CI 2.8-3.6 months), *P* = 0.725}, there was a trend towards longer median OS in the CHT group [15.5 months (95% CI 8.0-23.0 months) versus 5.3 months (95% CI 0-12.5 months), *P* = 0.052]. However, in multivariable analysis, type of first-line regimen (CHT versus sorafenib) was not associated with OS. ORR in patients receiving ICIs (*n* = 7) was 29%.

**Conclusions:**

In patients with cHCC-CCA, OS, progression-free survival, ORR, and disease control rate were not significantly different between individuals receiving CHT and patients receiving nCHT. Immunotherapy may be effective in a subset of patients. Prospective studies are needed to identify optimal systemic treatment regimens in cHCC-CCA.

## Highlights

• There is no optimal systemic treatment regimen for patients with cHCC-CCA.
• In this retrospective, multicenter cohort we describe clinical characteristics and outcome of 101 cHCC-CCA patients.
• Whereas PFS, ORR, and DCR were similar, OS trended to be longer in patients treated with first-line CHT vs. non-cytotoxic CHT (mainly sorafenib), but type of first-line regimen (CHT versus sorafenib) was not associated with OS in multivariable analysis.
• Treatment with ICIs may be effective in a subset of cHCC-CCA patients (ORR of 29%).

## Introduction

Primary liver cancer is the sixth most common malignant tumor and the third most common cause of cancer-related death globally.^1^ Whereas hepatocellular carcinoma (HCC) and intrahepatic cholangiocarcinoma (iCCA) represent the majority of cases, combined hepatocellular-cholangiocarcinoma (cHCC-CCA) accounts for only up to 5% of primary liver tumors.^1^^,^^2^ Per latest definition, classical cHCC-CCA requires the unequivocal exhibition of both cholangiocytic and hepatocytic differentiation within the same tumor on routine histopathology with hematoxylin–eosin (H&E) staining, regardless of the percentage of each component.^2^^,^^3^ Risk factors linked to the development of cHCC-CCA are largely unknown but may be similar to those reported for HCC.^4^ Resection is the only curative treatment option, but recurrence rate is high, and many patients eventually require systemic therapy during the course of the disease.^4^^,^^5^ Given the low prevalence of cHCC-CCA, difficulties with diagnosis, and the exclusion of patients with cHCC-CCA from clinical trials, there is no clear consensus on the optimal systemic treatment regimen.^4^ Platinum-based chemotherapy (CHT) and the tyrosine kinase inhibitor (TKI) sorafenib are frequently used,^4^ but the evidence level is low, as data were mainly derived from case series and small retrospective studies.^6^, ^7^, ^8^, ^9^, ^10^ Data on immunotherapy with immune checkpoint inhibitors (ICIs), which have proven efficacy in HCC^11^ and CCA,^12^ are lacking in patients with cHCC-CCA.^4^

In this retrospective, European multicenter study, we describe clinical characteristics and outcome of patients with histologically diagnosed cHCC-CCA, with a special focus on patients receiving palliative systemic therapy, including ICIs.

## Methods

### Study design

In this European multicenter study, patients with histologically proven cHCC-CCA, who were treated at four institutions in Austria and Germany between April 2003 and June 2022, were retrospectively included. The retrospective analysis of the data was approved by the local ethics committee of the Medical University of Vienna. Consent forms were waived due to the retrospective character of the study.

### Patients and definitions

Eligible patients were adults (>18 years) with histologically proven cHCC-CCA. Patients with other primary liver tumors (e.g. HCC, CCA, fibrolamellar carcinoma), hepatic metastasis due to other primary malignancies, missing or incomplete histological reports, and patients with insufficient records were excluded from this study. Patient characteristics, laboratory parameters (within 90 days before baseline), tumor characteristics, information on previous/current treatments, and Eastern Cooperative Oncology Group performance status (ECOG PS) were collected from the clinical documentation system. Diagnosis of cHCC-CCA was based on pathological assessments of biopsy samples (*n* = 52, 52%) and samples from surgical procedures (*n* = 49, 49%). Different types of first-line treatments were categorized as follows: surgical treatment (resection, liver transplantation), local ablation (radiofrequency/microwave ablation, percutaneous ethanol instillation), locoregional treatment [transarterial chemoembolization (TACE), SIRT, radiation], palliative systemic therapy, and best supportive care (BSC). Baseline was defined as date of histological diagnosis (overall cohort) and start of systemic therapy (palliative systemic therapy cohort), respectively.

### Palliative systemic therapy cohort

Patients with cHCC-CCA receiving palliative systemic therapy were assigned into a cytotoxic CHT versus non-cytotoxic CHT (nCHT) group. The CHT group included all conventional cytotoxic chemotherapies (i.e. platinum and non-platinum based chemotherapies) whereas TKIs and ICIs were included in the nCHT group. Decisions guiding the choice for type of first-line treatment were made in multidisciplinary tumor boards and were based on histological results, guidelines for HCC and CCA, and local therapy standards. All patients receiving palliative systemic therapy before 2007 (the year of sorafenib approval) received cytotoxic CHT (*n* = 4). Patients who received classical adjuvant CHT after surgical resection or systemic therapy in combination with TACE for liver-limited disease were excluded from this cohort. One patient who received CHT after R1 resection was included. Three patients who received systemic therapy in combination with locoregional treatment were not included in either group. One of these patients, however, received conventional CHT after having progressed under the combination treatment and was therefore included at the time of CHT start.

### Efficacy and definition of main outcomes

Duration of systemic therapy was defined as time from treatment initiation until date of treatment stop or death. Overall survival (OS) was defined as the time from diagnosis (overall cohort) or systemic therapy start (systemic therapy cohort) until date of death or last contact; patients who were alive/lost to follow-up were censored at last contact. Progression-free survival (PFS) was defined as time from systemic therapy start until date of radiological progression or date of death/last follow-up, whatever came first; patients who were alive/lost to follow-up without progression were censored at last contact. Overall response rate (ORR) was defined as the proportion of patients with complete response (CR) or partial response (PR), whereas disease control rate (DCR) was defined as the proportion of patients with CR, PR, or stable disease (SD) as best objective response. Best objective response was assessed according to the modified Response Evaluation Criteria in Solid Tumors (mRECIST).^13^

### Statistics

Statistical analyses were carried out using IBM SPSS Statistics 27 (SPSS Inc., Armonk, NY) and GraphPad Prism 9 (GraphPad Software, La Jolla, CA). Sankey plots were created using SankeyMATIC (https://sankeymatic.com/about/). Continuous variables were reported as mean ± standard deviation or median (interquartile range), and categorical variables were shown as numbers (*n*) and proportions (%) of patients. Comparisons of proportions and of continuous variables were carried out by chi-square test and unpaired Student’s *t*-test, respectively. Median estimated follow-up was calculated by the reverse Kaplan–Meier method. The Kaplan–Meier method was used to calculate survival curves and comparison was carried out by the log-rank test. In patients with incomplete dates, i.e. available month and year but missing day, the 15th of the respective month was used for calculations. Multivariable analysis was carried out by Cox regression analysis and variables with a *P* value <0.05 in univariable analysis were included. A two-sided *P* value ≤0.05 was considered statistically significant.

## Results

### Patient characteristics (overall cohort)

In total, 101 patients with histologically proven cHCC-CCA were treated at four institutions between April 2003 and June 2022. Most patients were male (*n* = 70, 69%) with a mean age of 64.6 ± 10.6 years. Cirrhosis was present in 44 (44%) patients, predominantly with non-viral etiology (*n* = 81, 80%). The majority of patients had a preserved liver function (Child–Pugh score A: *n* = 66, 65%), but an advanced stage cHCC-CCA (Barcelona Clinic Liver Cancer B-D: *n* = 70, 69%). The most common type of first-line treatment was a surgical procedure (*n* = 54, 53%), followed by locoregional therapy (including local ablation) (*n* = 24, 24%), systemic therapy (*n* = 14, 14%), and BSC (*n* = 7, 7%). Median estimated follow-up was 38.5 months [95% confidence interval (CI) 31.3-45.6 months]. Detailed patient characteristics are shown in Table 1.

**Table 1:** Baseline characteristics of all patients with cHCC-CCA

	All patients *N* = 101
	*n* (%) and median (IQR)/mean ± SD
Age, years	64.6 ± 10.6
Range	23.3-84.8
>65	55 (55)
Sex	
Male	70 (69)
Female	31 (31)
Cirrhosis	
Yes	44 (44)
No	57 (56)
Etiology of liver disease	
Viral	20 (20)
HBV	12 (12)
HCV	8 (8)
Non-viral	81 (80)
Child–Pugh score, points^a^	5 (5-7)
A	66 (65)
B	19 (19)
C	3 (3)
ALBI grade, points^b^	-2.7 (-3.1 to -2.1)
1	46 (46)
2	33 (33)
3	10 (10)
ECOG PS^b^	
0	56 (55)
≥1	39 (39)
Macrovascular invasion^c^	
Yes	12 (12)
No	85 (85)
Extrahepatic metastases	
Yes	11 (11)
No	90 (89)
BCLC stage^d^	
A	25 (25)
B	10 (10)
C	56 (55)
D	4 (4)
UICC (TNM) stage^e^	
≤IIIa	65 (64)
≥IIIb	34 (34)
Tumor grading^f^	
G1/G2 (well to moderately differentiated)	52 (52)
G3 (poorly differentiated)	31 (31)
AFP, IU/ml^f^	33.9 (4.8-1370)
<200	51 (51)
≥200	32 (32)
CA 19-9, kU/l^g^	44 (13.6-81.2)
<200	58 (58)
≥200	10 (10)
Type of first-line treatment^c^	
Surgical	54 (53)
Resection	49 (49)
LTx	5 (5)
Local ablation	4 (4)
RFA/MWA	1 (1)
PEI	3 (3)
Locoregional	20 (20)
TACE^h^	16 (16)
SIRT	1 (1)
Radiation	3 (3)
Systemic therapy	14 (14)
BSC	7 (7)

AFP, α-fetoprotein; ALBI grade, albumin-bilirubin grade; BCLC, Barcelona Clinic Liver Cancer; BSC, best supportive care; CA 19-9, carbohydrate-antigen 19-9; cHCC-CCA, combined hepatocellular-cholangiocarcinoma; ECOG PS, Eastern Cooperative Oncology Group performance status; HBV, hepatitis B virus; HCV, hepatitis C virus; IQR, interquartile range; LTx, liver transplantation; MWA, microwave ablation; PEI, percutaneous ethanol instillation; RFA, radiofrequency ablation; SD, standard deviation; SIRT, selective internal radiotherapy; TACE, transarterial chemoembolization; TNM, tumor–node–metastasis; UICC, International Union Against Cancer.

a Missing values in *n* = 13 patients.

b Missing values in *n* = 12 patients.

c Missing values in *n* = 4 patients.

d Missing values in *n* = 6 patients.

e Missing values in *n* = 2 patients.

f Missing values in *n* = 18 patients.

g Missing values in *n* = 33 patients.

h TACE + bevacizumab (*n* = 1), TACE + sorafenib (*n* = 1), TACE + nivolumab (*n* = 1).

### Outcome of patients with cHCC-CCA (overall cohort)

**Figure 1:**
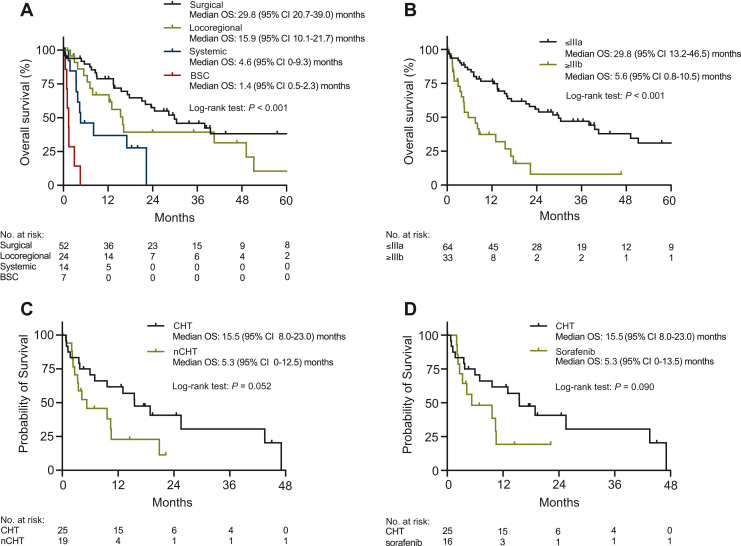
**Kaplan–Meier survival curves of patients with cHCC-CCA.** Overall survival of all cHCC-CCA patients according to first-line treatment (A) and International Union Against Cancer (UICC) [TNM (tumor–node–metastasis)] stage (B). Overall survival of patients treated with palliative systemic therapy receiving CHT versus nCHT (C) and CHT versus sorafenib (D) in systemic first line. BSC, best supportive care; cHCC-CCA, combined hepatocellular-cholangiocarcinoma; CHT, chemotherapy; CI, confidence interval; nCHT, non-cytotoxic chemotherapy; OS, overall survival.

Median OS was 17.2 months (95% CI 10.4-24 months). Patients who underwent surgery as first-line treatment had a significantly better outcome compared with patients receiving locoregional therapy (e.g. TACE, ablation), systemic therapy or BSC [surgery: 29.8 months (95% CI 20.7-39.0 months) versus locoregional therapy: 15.9 months (95% CI 10.1-21.7 months) versus systemic therapy: 4.6 months (95% CI 0-9.3 months) versus BSC: 1.4 months (95% CI 0.5-2.3 months); *P* < 0.001] (Figure 1A). Similarly, median OS significantly differed according to International Union Against Cancer (UICC) [TNM (tumor–node–metastasis)] stages [≤IIIa: 29.8 months (13.2-46.5 months) versus ≥IIIb: 5.6 months (0.8-10.5 months); *P* < 0.001] (Figure 1B). In multivariable analyses, only type of first-line treatment [BSC: hazard ratio (HR) 6.95 (95% CI 1.21-40.07), *P* = 0.030] remained significantly associated with reduced OS, independently of age, performance status, CA 19-9, and UICC (TNM) stage (Table 2).

**Table 2:** Uni- and multivariable Cox regression analyses of prognostic factors for overall survival (all patients)

	Univariable			Multivariable		
	HR	95% CI	*P* value	HR	95% CI	*P* value
Sex				—	—	—
Male	1					
Female	1.07	0.63-1.83	0.795			
Age, years			**0.016**^a^	1	0.80-3.04	0.194
<65	1			1.56		
>65	1.88	1.12-3.12				
Type of first-line treatment						
Surgical	1			1		
Non-surgical	1.90	1.10-3.27	**0.022**	1.62	0.73-3.57	0.235
BSC	18.06	6.62-49.27	**<0.001**	6.95	1.21-40.07	**0.030**
Etiology				—	—	—
Viral	1					
Non-viral	0.67	0.36-1.24	0.204			
CPS stage				—	—	—
A	1					
B	1.17	0.60-2.27	0.653			
C	0.76	0.18-3.22	0.708			
ALBI grade				—	—	—
1	1					
2	1.17	0.66-2.09	0.593			
3	1.92	0.79-4.71	0.152			
ECOG PS						
0	1			1		
≥1	2.02	1.19-3.41	**0.009**	1.46	0.66-3.26	0.351
Macrovascular invasion				—	—	—
Yes	1					
No	2.31	1.15-4.64	**0.018**			
Extrahepatic metastases				—	—	—
Yes	1					
No	2.46	1.09-5.59	**0.031**			
AFP, IU/ml				—	—	—
<200	1					
≥200	1.67	0.95-2.95	0.077			
CA 19-9, kU/l				1	0.28-3.79	0.966
<200	1			1.03		
≥200	3.09	1.33-7.21	**0.009**			
Tumor grading^b^				—	—	—
G1/G2	1					
G3	1.49	0.83-2.69	0.184			
UICC (TNM) stage						
≤IIIa	1			1		
≥IIIb	3.23	1.84-5.69	**<0.001**	1.93	0.91-4.10	0.088

AFP, α-fetoprotein; ALBI grade, albumin-bilirubin grade; BSC, best supportive care; CA 19-9, carbohydrate-antigen 19-9; CI, confidence interval; CPS, Child-Pugh score; ECOG PS, Eastern Cooperative Oncology Group performance status; HR, hazard ratio; TNM, tumor–node–metastasis; UICC, International Union Against Cancer.

a Significant values are marked bold.

b G1/G2: well to moderately differentiated; G3: poorly differentiated.

### Outcome of patients with cHCC-CCA treated with palliative systemic therapy

In total, 44 (44%) patients were treated with palliative systemic therapy for a median duration of 2.5 months (95% CI 1.5-3.6 months). Of those, 25 (57%) patients received cytotoxic CHT and 19 (43%) patients were treated with nCHT in systemic first line. A considerable proportion of patients [CHT: *n* = 14 (56%), nCHT: *n* = 6 (32%)] received at least one subsequent therapy. Prior and subsequent anticancer treatments as well as baseline characteristics were similar between both groups. Only the number of patients receiving subsequent cytotoxic CHT was higher in the CHT [*n* = 10 (40%)] than in the nCHT group [*n* = 1 (5%), *P* = 0.013] (Supplementary Table S1 and Supplementary Figure S1, available at https://doi.org/10.1016/j.esmoop.2023.100783). Type of systemic first-line therapy is displayed in Supplementary Table S2, available at https://doi.org/10.1016/j.esmoop.2023.100783.

Whereas we did not observe a significant difference in PFS [CHT: 3.0 months (95% CI 1.4-4.6 months) versus nCHT: 3.2 months (95% CI 2.8-3.6 months), *P* = 0.725], there was a strong trend towards longer OS in the CHT group [CHT: 15.5 months (95% CI 8.0-23.0 months) versus nCHT: 5.3 months (95% CI 0-12.5 months), *P* = 0.052] (Figure 1C). ORR (CHT 8% versus nCHT 5%, *P* = 1.000) and DCR (CHT 24% versus nCHT 21%, *P* = 1.000) were similar between both groups (Table 3).

**Table 3:** Efficacy results of cHCC-CCA patients receiving palliative cytotoxic chemotherapy (CHT) and non-cytotoxic chemotherapy (nCHT) as systemic first-line treatment

	CHT *N* = 25	nCHT *N* = 19	*P* value
	*n* (%)	*n* (%)	
Best objective response			0.751
CR	—	—	
PR	2 (8)	1 (5)	
SD	4 (16)	3 (16)	
PD	11 (44)	6 (32)	
NE	8 (32)	9 (47)	
ORR (CR + PR)	2 (8)	1 (5)	1.000
DCR (CR + PR + SD)	6 (24)	4 (21)	1.000
Median OS, months	15.5 (95% CI 8.0-23.0)	5.3 (95% CI 0-12.5)	0.052
Median PFS, months	3.0 (95% CI 1.4-4.6)	3.2 (95% CI 2.8-3.6)	0.725

cHCC-CCA, combined hepatocellular-cholangiocarcinoma; CHT, cytotoxic chemotherapy; CI, confidence interval; CR, complete response; DCR, disease control rate; nCHT, non-cytotoxic chemotherapy; NE, not evaluable; ORR, overall response rate; OS, overall survival; PD, progressive disease; PFS, progression-free survival; PR, partial response; SD, stable disease.

We next compared the outcome of cHCC-CCA patients treated with CHT (*n* = 25) versus those treated with sorafenib (*n* = 16) in systemic first line, and observed no differences regarding baseline characteristics (Supplementary Table S1, available at https://doi.org/10.1016/j.esmoop.2023.100783). Patients treated with CHT had a numerically higher ORR (8% versus 0%) and DCR (24% versus 19%) than patients receiving sorafenib. PFS, however, was similar between both groups [CHT: 3.0 months (95% CI 1.4-4.6 months) versus sorafenib: 3.1 months (95% CI 2.7-3.5 months), *P* = 0.843] (Supplementary Table S3, available at https://doi.org/10.1016/j.esmoop.2023.100783). Median OS was more favorable in the CHT compared with the sorafenib group [CHT: 15.5 months (95% CI 8.0-23.0 months) versus sorafenib: 5.3 months (95% CI 0-13.5 months), *P* = 0.090] (Figure 1D), but in multivariable Cox regression analysis, only age [age >65 years: HR 10.95 (95% CI 1.36-88.36), *P* = 0.025] and albumin-bilirubin (ALBI) grade [ALBI 3: HR 11.81 (95% CI 1.11-125.85), *P* = 0.041] remained significantly associated with reduced OS (Supplementary Table S4, available at https://doi.org/10.1016/j.esmoop.2023.100783).

### Outcome of patients with cHCC-CCA treated with immunotherapy

Of seven patients receiving ICIs during their course of therapy, three (43%) patients received atezolizumab plus bevacizumab, two (29%) patients were treated with nivolumab alone, and one (14%) patient each received pembrolizumab alone and nivolumab in combination with TACE, respectively (Table 4). ICIs were administered in three (43%) patients in systemic first line, in one (14%) patient in second line, and in three (43%) patients in later lines. Two (29%) patients had partial response and one (14%) patient had SD, accounting for an ORR of 29% and a DCR of 43%. Median PFS and OS from start of immunotherapy were 4.1 months (95% CI 2.4-5.8 months) and 17.8 (95% CI 16.0-19.5 months), respectively.

**Table 4:** Description and efficacy results of cHCC-CCA patients treated with immune checkpoint inhibitors

	Patients receiving IT (*N* = 7)
Type of ICI, *n* (%)	
Atezolizumab + bevacizumab	3 (43)
Nivolumab	2 (29)
Nivolumab + TACE	1 (14)
Pembrolizumab	1 (14)
Line of systemic treatment, *n* (%)	
First-line	3 (43)
Second-line	1 (14)
Later lines	3 (43)
Best overall response, *n* (%)	
CR	—
PR	2 (29)
SD	1 (14)
PD	1 (14)
NE	3 (43)
ORR (CR + PR), *n* (%)	2 (29)
DCR (CR + PR + SD), *n* (%)	3 (43)
Median OS, months	17.8 (95% CI 16.0-19.5)
Median PFS, months	4.1 (95% CI 2.4-5.8)

cHCC-CCA, combined hepatocellular-cholangiocarcinoma; CR, complete response; DCR, disease control rate; ICI, immune checkpoint inhibitor; IT, immunotherapy; NE, not evaluable; ORR, overall response rate; OS, overall survival; PD, progressive disease; PFS, progression-free survival; PR, partial response; SD, stable disease; TACE, transarterial chemoembolization.

## Discussion

Of 101 patients with histologically diagnosed cHCC-CCA, a total of 77% of patients received surgical or locoregional therapies as primary treatment. In patients who received palliative systemic therapy during their course of treatment, there was no difference in ORR, DCR, and PFS, but a strong trend towards better OS in cytotoxic CHT-treated patients compared with the nCHT/sorafenib group. In multivariable analysis, however, type of first-line regimen (CHT versus sorafenib) was not associated with OS. ICI-based regimens administered in different treatment lines showed promising efficacy (ORR, 29%). To the best of our knowledge, this is one of the largest European cohorts of cHCC-CCA patients treated with systemic therapy published to date and the only study reporting on cHCC-CCA patients receiving ICIs.

When comparing sorafenib with cytotoxic CHT, our results are partly in line with data from the United States,^7^ Japan,^6^ Korea,^8^ and France.^10^ In a retrospective, monocentric United States cohort of patients with cHCC-CCA receiving systemic therapy, DCR was lowest in those treated with sorafenib (*n* = 7) compared with patients treated with gemcitabine plus platinum (*n* = 41) or gemcitabine ± fluoropyrimidine (*n* = 16) (DCR, 20% versus 78.4% versus 38.5%). Survival was also shorter in the sorafenib-treated subgroup (median OS, 9.6 versus 11.5 versus 11.7).^7^ Similarly, in a retrospective, Japanese multicenter study including 36 patients with cHCC-CCA treated with systemic therapies, patients receiving sorafenib (*n* = 5) had a shorter survival than those treated with fluorouracil plus cisplatin (*n* = 11) and gemcitabine plus cisplatin (*n* = 12) (median OS, 3.5 versus 11.9 versus 10.2 months).^6^ Hence, both studies attested sorafenib only limited efficacy. The conclusions of both studies, however, are limited by the very small number of patients in the sorafenib group and were recently challenged by two larger studies.^8^^,^^10^ A retrospective, single-center study from Korea found no significant differences in survival outcomes between sorafenib- (*n* = 62) and cytotoxic CHT-treated (*n* = 37) patients (median PFS, 4.2 versus 2.9 months; median OS, 10.7 versus 10.6 months).^8^ Similar results were obtained in a French retrospective multicenter study, where OS and PFS of cHCC-CCA patients (*n* = 25) treated with TKIs did not significantly differ from patients (*n* = 54) receiving platinum-based regimens (median PFS, 2.8 versus 4.1 months; median OS, 8.3 versus 11.9 months).^10^

Patients with cHCC-CCA may also be candidates for immunotherapy, as ICIs are effective in both HCC^11^ and CCA.^12^ Only recently, a subgroup of cHCC-CCA was identified showing features of a sustained intratumoral immune response as well as activated gene signatures predicting response to immunotherapy in HCC. This subclass was associated with improved survival and may benefit from ICIs.^14^ Clinical data on immunotherapy in this rare liver tumor are limited, however, to a case report of a patient with metastatic cHCC-CCA who achieved a complete remission on third-line treatment with pembrolizumab.^15^ In our cohort, a total of seven patients received ICI-based systemic therapies in different treatment lines, and achieved a promising ORR of 29% and a DCR of 43%. These data strongly suggest that ICI-based regimens should be further evaluated in patients with cHCC-CCA.

Limitations of this study include the retrospective nature with all its known potential shortcomings, the long recruitment period potentially influencing our results by changing clinical practices, as well as the large heterogeneity among included patients. Despite being one of the largest cohorts reporting on palliative systemic therapy (and the largest reporting on immunotherapy) in patients with cHCC-CCA published so far, the sample size is still small. Thus, confirmation of these results in large prospective trials would be desirable, but such studies may be difficult to conduct due to the low prevalence and difficulties in diagnosing cHCC-CCA.

In conclusion, type of first-line treatment was independently associated with worse OS in patients with cHCC-CCA. In the subgroup of patients who received palliative systemic therapy, there was a trend towards longer OS in cytotoxic CHT-treated patients (versus nCHT/sorafenib group), whereas other efficacy endpoints (PFS, ORR, and DCR) were not different. In multivariable analysis, type of first-line regimen (CHT versus sorafenib) was not associated with OS. Similarly to HCC and CCA, a proportion of patients with cHCC-CCA seem to benefit from ICIs.

## Funding

None declared.

## Disclosure

**BS** received travel support from AbbVie, Ipsen, and Gilead. **MT** received speaker fees from Bristol-Myers Squibb (BMS), Falk Foundation, Gilead, Intercept, and Merck Sharp & Dohme (MSD); advisory board fees from AbbVie, Albireo, Boehringer Ingelheim, BiomX, Falk Pharma GmbH, GENFIT, Gilead, HighTide, Intercept, Janssen, MSD, Novartis, Phenex, Regulus, and Shire; travel grants from AbbVie, Falk, Gilead, and Intercept; and research grants from Albireo, CymaBay, Falk, Gilead, Intercept, MSD, and Takeda. He is also coinventor of patents on the medical use of norUDCA filed by the Medical University of Graz. **FF** received travel support from AbbVie and Novartis, and speaker fees from AbbVie and MSD. **AW** received compensations as a member of scientific advisory boards for BMS, Wako and Sanofi. He served as a speaker for Leo Pharma, Eisai, Ipsen, and Roche and received travel support from Merck and Servier. **AV** served as consultant for Roche, Bayer, Lilly, BMS, Eisai, and Ipsen, and received speaking fees from Roche, Bayer, Lilly, BMS, Eisai, and Ipsen. He is also an investigator for Roche, Bayer, Lilly, BMS, Eisai, and Ipsen. **MP** is an investigator for Bayer, BMS, Eisai, Ipsen, Lilly, and Roche; he received speaker honoraria from Bayer, BMS, Eisai, Lilly, MSD, and Roche; he is a consultant for Bayer, BMS, Eisai, Ipsen, Lilly, MSD, and Roche; he received travel support from Bayer, BMS, and Roche. All other authors have declared no conflicts of interest.
