# Supplementary material for: Clinical characteristics and outcome of patients with combined hepatocellular-cholangiocarcinoma—a European multicenter cohort
Source: ESMO Open. 2023 Feb 6;8(1):100783. doi: 10.1016/j.esmoop.2023.100783 (PMC10024130; doi:10.1016/j.esmoop.2023.100783)
Supplement: Supplementary data [file mmc1.docx]

| **Supplemental Table 1. Baseline characteristics of cHCC-CCA patients receiving palliative systemic therapy** | | | | | | |
| --- | --- | --- | --- | --- | --- | --- |
|  | | | | | | |
|  | **CHT vs. nCHT** | | | **CHT vs. sorafenib** | | |
|  | **CHT**  **n=25** | **nCHT**  **n=19** | **p-value** | **CHT**  **n=25** | **sorafenib n=16** | **p-value** |
|  | **N (%) and median (IQR) / mean ± SD** | | | | | |
| **Age,** years  Range | 65.2±9.6  41.2-81.8 | 65.2±11.7  31.2-82.8 | 0.999 | 65.2±9.6  41.2-81.8 | 67.1±7.6  51.3-82.8 | 0.493 |
| **Age**, >65 years  Yes  No | 14 (56%)  11 (44%) | 12 (63%)  7 (37%) | 0.632 | 14 (56%)  11 (44%) | 11 (69%)  5 (31%) | 0.414 |
| **Sex**  Male  Female | 14 (56%)  11 (44%) | 16 (84%)  3 (16%) | 0.058 | 14 (56%)  11 (44%) | 13 (81%)  3 (19%) | 0.176 |
| **Etiology of liver disease**  Viral  Non-viral | 4 (16%)  21 (84%) | 3 (16%)  16 (84%) | 1.000 | 4 (16%)  21 (84%) | 1 (6%)  15 (94%) | 0.632 |
| **CPS**, points ^1^ | 6 (5-7) | 6 (5-8) | 0.503 | 6 (5-7) | 6 (5-8) | 0.587 |
| **CPS stage** ^1^  A  B  C | 12 (48%)  6 (24%)  - | 9 (47%)  7 (37%)  1 (5%) | 0.600 | 12 (48%)  6 (24%)  - | 8 (50%)  5 (31%)  1 (6%) | 0.706 |
| **ALBI,** points ^1^ | -2.38  ([-2.94]- [-1.83]) | -1.92  ([-2.69]- [-1.36]) | 0.153 | -2.38  ([-2.94]- [-1.83]) | -1.97  ([-2.69]-  [-1.36]) | 0.251 |
| **ALBI grade** ^1^  1  2  3 | 7 (28%)  9 (36%)  2 (8%) | 5 (26%)  7 (37%)  5 (26%) | 0.420 | 7 (28%)  9 (36%)  2 (8%) | 4 (25%)  6 (38%)  4 (25%) | 0.517 |
| **ECOG PS** ^2^  0  ≥1 | 10 (40%)  14 (56%) | 6 (32%)  13 (68%) | 0.497 | 10 (40%)  14 (56%) | 3 (19%)  13 (81%) | 0.130 |
| **Macrovascular invasion** ^2^  Yes  No | 8 (32%)  16 (64%) | 2 (11%)  17 (89%) | 0.145 | 8 (32%)  16 (64%) | 2 (12%)  14 (88%) | 0.263 |
| **Extrahepatic metastases**  Yes  No | 14 (56%)  11 (44%) | 15 (79%)  4 (21%) | 0.112 | 14 (56%)  11 (44%) | 12 (75%)  4 (25%) | 0.218 |
| **BCLC stage** ^2^  B  C | 5 (20%)  19 (76%) | 1 (5%)  18 (95%) | 0.205 | 5 (20%)  19 (76%) | -  16 (100%) | 0.071 |
| **UICC (TNM) stage**  <IIIa  >IIIb | 11 (44%)  14 (56%) | 4 (21%)  15 (79%) | 0.112 | 11 (44%)  14 (56%) | 4 (25%)  12 (75%) | 0.218 |
| **AFP** IU/mL ^3^ | 11.5  (2.1-3841) | 347.7  (5.6-6053.2) | 0.313 | 11.5  (2.1-3841) | 66  (5-6053.2) | 0.495 |
| **AFP** IU/mL ^3^  <200  ≥200 | 11 (44%)  5 (20%) | 8 (42%)  10 (53%) | 0.154 | 11 (44%)  5 (20%) | 8 (50%)  7 (44%) | 0.379 |
| **CA 19-9** kU/L ^4^ | 78  (16-145.9) | 30  (12-45) | 0.234 | 78  (16-145.9) | 30  (11-59.5) | 0.236 |
| **CA 19-9** kU/L ^4^  <200  ≥200 | 12 (48%)  3 (12%) | 13 (68%)  1 (5%) | 0.598 | 12 (48%)  3 (12%) | 11 (69%)  1 (6%) | 0.605 |
| **Prior surgical treatment**  Yes  No | 9 (36%)  16 (64%) | 10 (53%)  9 (47%) | 0.270 | 9 (36%)  16 (64%) | 10 (63%)  6 (37%) | 0.097 |
| **Prior ablation**  Yes  No | 2 (8%)  23 (92%) | 2 (11%)  17 (89%) | 1.000 | 2 (8%)  23 (92%) | 2 (13%)  14 (87%) | 0.637 |
| **Prior TACE**  Yes  No | 3 (12%)  22 (88%) | 6 (32%)  13 (68%) | 0.144 | 3 (12%)  22 (88%) | 6 (38%)  10 (62%) | 0.119 |
| **Prior radiation**  Yes  No | 2 (8%)  23 (92%) | 1 (5%)  18 (95%) | 1.000 | 2 (8%)  23 (92%) | 1 (6%)  15 (94%) | 1.000 |
| **Prior SIRT**  Yes  No | 1 (4%)  24 (96%) | 2 (11%)  17 (89%) | 0.570 | 1 (4%)  24 (96%) | 2 (13%)  14 (87%) | 0.550 |
| **Type of second-line treatment**  CHT  TKI  IT  Any local therapy*  None | 8 (32%)  3 (12%)  1 (4%)  2 (8%)  11 (44%) | 1 (5%)  2 (11%)  1 (5%)  2 (11%)  13 (68%) | 0.226 | 8 (32%)  3 (12%)  1 (4%)  2 (8%)  11 (44%) | 1 (6%)  2 (12.5%)  -  2 (12.5%)  11 (69%) | 0.270 |
| **Number of all subsequent anti-cancer therapies**  1  2  3  4  5  None | 6 (24%)  4 (16%)  -  3 (12%)  1 (4%)  11 (44%) | 2 (11%)  3 (16%)  1 (5%)  -  -  13 (68%) | 0.255 | 6 (24%)  4 (16%)  -  3 (12%)  1 (4%)  11 (44%) | 2 (12%)  3 (19%)  -  -  -  11 (69%) | 0.417 |
| **Subsequent CHT**  Yes  No | 10 (40%)  15 (60%) | 1 (5%)  18 (95%) | **0.013** | 10 (40%)  15 (60%) | 1 (6%)  15 (94%) | **0.028** |
| **Subsequent TKI**  Yes  No | 4 (16%  21 (84% | 4 (21%)  15 (79%) | 0.710 | 4 (16%  21 (84% | 3 (19%)  13 (81%) | 1.000 |
| **Subsequent IT**  Yes  No | 3 (12%)  22 (88%) | 2 (10%)  17 (90%) | 1.000 | 3 (12%)  22 (88%) | 1 (6%)  15 (94%) | 1.000 |
| **Subsequent local therapy ***  Yes  No | 5 (20%)  20 (80%) | 2 (10%)  17 (90%) | 0.680 | 5 (20%)  20 (80%) | 2 (12%)  14 (88%) | 0.685 |
| *Missing values in ^1^ n=7 patients (CHT), n=2 patients (nCHT, sorafenib); ^2^ n=1 patient (CHT); ^3^ n=9 patients (CHT), n=1 patient (nCHT, sorafenib); ^4^ n=10 patients (CHT), n=5 patients (nCHT), n=4 patients (sorafenib)*  ** local therapy includes: ablation, TACE, radiation, SIRT, surgical resection* | | | | | | |
| **Abbreviations:** AFP, α-fetoprotein; ALBI grade, Albumin-Bilirubin grade; BCLC, Barcelona Clinic Liver Cancer; BSC, best supportive care; CA 19-9, carbohydrate-antigen 19-9; cHCC-CCA, combined hepatocellular-cholangiocarcinoma; CHT, cytotoxic chemotherapy; CPS, Child-Pugh score; ECOG PS, Eastern Cooperative Oncology Group performance status; IT, immunotherapy; nCHT, non-cytotoxic chemotherapy; SIRT, selective internal radiotherapy; TACE, transarterial chemoembolization; TKI, tyrosine-kinase inhibitor; UICC, Union for International Cancer Control. | | | | | | |

| **Supplemental Table 2. Types of palliative systemic first-line regimens in patients with cHCC-CCA** | |
| --- | --- |
|  | |
| **First-line Treatment** | **Number of patients**  **n=44** |
| **Tyrosine kinase inhibitor**  Lenvatinib  Sorafenib | **17 (38.6%)**  1 (2.3%)  16 (36.3%) |
| **Platinum based chemotherapy**  Gemcitabine/Cisplatin  Gemcitabine/Oxaliplatin  Gemcitabine/Oxaliplatin/Bevacizumab | **17 (38.6%)**  14 (31.7%)  2 (4.6%)  1 (2.3%) |
| **Non-platinum based chemotherapy**  5-FU  5-FU  Capecitabine  Gemcitabine | **8 (18.2%)**  1 (2.3%)  1 (2.3%)  1 (2.3%)  5 (11.3%) |
| **Immunotherapy**  Atezolizumab/Bevacizumab  Nivolumab | **2 (4.6%)**  1 (2.3%)  1 (2.3%) |
|  | |
| **Abbreviations:** cHCC-CCA, combined hepatocellular-cholangiocarcinoma; 5-FU, 5-Flourouracil. | |

| **Supplemental Table 3. Efficacy results of cHCC-CCA patients receiving palliative cytotoxic chemotherapy (CHT) vs. sorafenib as systemic first-line treatment** | | | | |
| --- | --- | --- | --- | --- |
|  | **CHT**  **n= 25** | | **Sorafenib**  **n= 16** |  |
|  | **n (%)** | **n (%)** | | **p-value** |
| **Best objective response**  CR  PR  SD  PD  NE | -  2 (8%)  4 (16%)  11 (44%)  8 (32%) | | -  -  3 (19%)  5 (31%)  8 (50%) | 0.468 |
| **ORR** (CR+PR) | 2 (8%) | | - | 1.000 |
| **DCR** (CR+PR+SD) | 6 (24%) | | 3 (19%) | 1.000 |
| **Median OS** | 15.5  (95%CI: 8.0-23.0) | | 5.3  (95%CI: 0-13.5) | 0.090 |
| **Median PFS** | 3.0  (95%CI: 1.4-4.6) | | 3.1  (95%CI: 2.7-3.5) | 0.843 |
| **Abbreviations:** cHCC-CCA, combined hepatocellular-cholangiocarcinoma; CHT, cytotoxic chemotherapy; CR, complete response; ORR, overall response rate; DCR, disease control rate; NE, not evaluable; nCHT, non-cytotoxic chemotherapy; OS, overall survival; PD, progressive disease; PFS, progression-free survival; PR, partial response; SD, stable disease. | | | | |

| **Supplemental Table 4. Uni- and multivariable Cox regression analysis of prognostic factors for overall survival in patients treated with palliative systemic therapy (CHT vs. sorafenib)** | | | | | | |
| --- | --- | --- | --- | --- | --- | --- |
|  | | | | | | |
|  | **Univariable** | | | **Multivariable** | | |
|  | **HR** | **95% CI** | **p-value** | **HR** | **95% CI** | **p-value** |
| **Type of first-line Treatment**  Sorafenib  CHT | 1  0.49 | 0.21-1.14 | 0.097 | 1  0.70 | 0.26-1.87 | 0.478 |
| **Sex**  Male  Female | 1  1.73 | 0.73-4.09 | 0.211 | - | - | - |
| **Age**  <65 years  >65 years | 1  5.97 | 1.95-18.29 | **0.002** | 1  10.95 | 1.36-88.36 | **0.025** |
| **Etiology**  Viral  Non-viral | 1  0.69 | 0.24-2.03 | 0.502 | - | - | - |
| **CPS stage**  A  B  C | 1  1.08  3.56 | 0.41-2.87  0.43-29.64 | 0.879  0.241 | - | - | - |
| **ALBI grade**  1  2  3 | 1  3.17  2.31 | 1.10-9.13  0.55-9.74 | **0.032**  0.255 | 1  2.44  11.81 | 0.83-7.19  1.11-125.85 | 0.106  **0.041** |
| **ECOG PS**  0  ≥1 | 1  2.36 | 0.97-5.70 | 0.057 | - | - | - |
| **Macrovascular invasion**  Yes  No | 1  1.14 | 0.49-2.65 | 0.767 | - | - | - |
| **Extrahepatic spread**  Yes  No | 1  1.47 | 0.63-3.42 | 0.371 | - | - | - |
| **AFP** IU/mL  <200  ≥200 | 1  1.54 | 0.63-3.80 | 0.348 | - | - | - |
| **CA 19-9** kU/L  <200  ≥200 | 1  0.64 | 0.08-4.96 | 0.671 | - | - | - |
| **UICC (TNM) stage**  ≤IIIa  ≥IIIb | 1  1.47 | 0.63-3.42 | 0.371 | - | - | - |
|  | | | | | | |
| **Abbreviations:** BSC, best supportive care; CI, confidence interval; CPS, Child-Pugh score; ECOG PS, Eastern Cooperative Oncology Group performance status; HR, hazard ratio; TNM, TNM classification of malignant tumors; UICC, Union for International Cancer Control. | | | | | | |

| **Supplemental Figure 1. Types of palliative systemic first- and second-line regimens in patients with cHCC-CCA treated with cytotoxic chemotherapy (CHT) (a) and non-cytotoxic chemotherapy (nCHT) (b)** |
| --- |
|  |
| **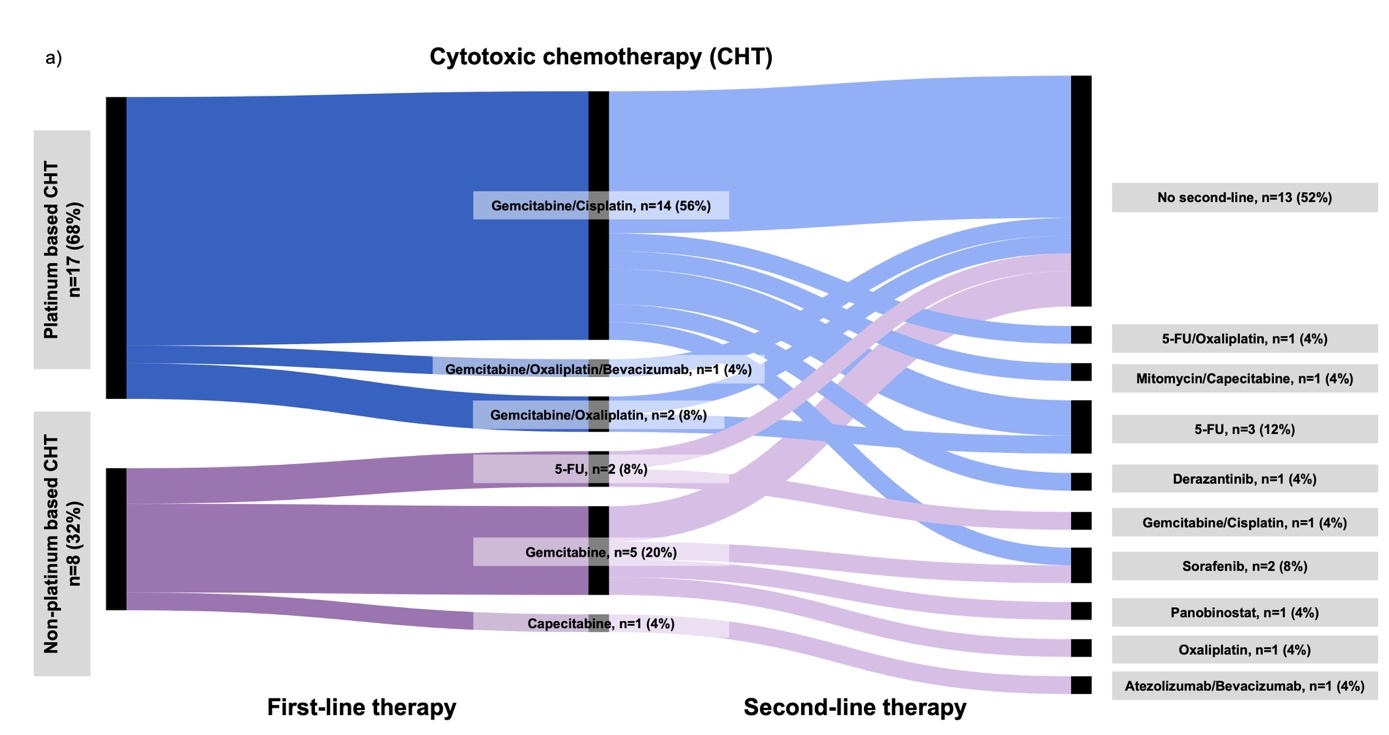**  **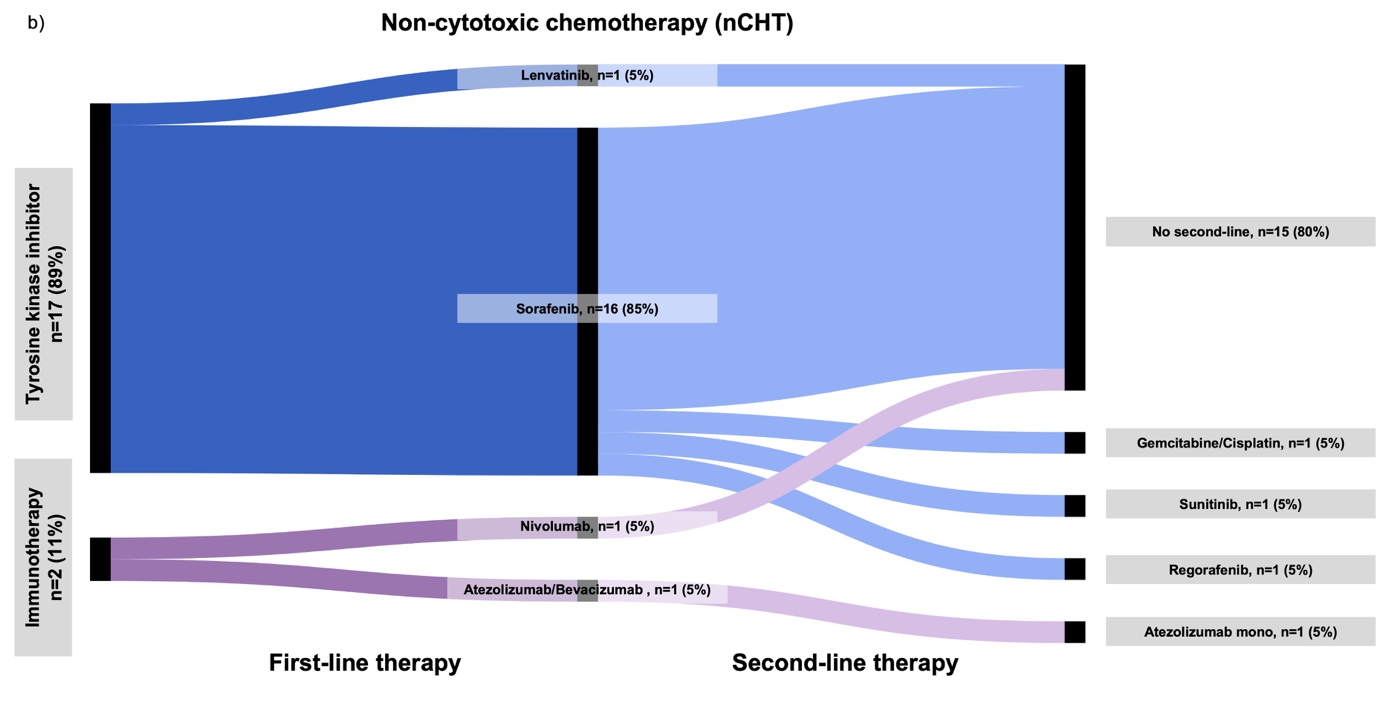** |
|  |
| **Abbreviations:** cHCC-CCA, combined hepatocellular-cholangiocarcinoma; CHT, cytotoxic chemotherapy; nCHT, non-cytotoxic chemotherapy; 5-FU, 5-Flourouracil. |
